# Cardioprotection in coronary artery bypass graft surgery: the impact of remote ischemic preconditioning on modulating LOX-1 and SOD-1 to counteract oxidative stress

**DOI:** 10.3389/fcvm.2024.1502326

**Published:** 2024-10-25

**Authors:** Cezar-Dumitrel Luca, Alexandra Boieriu, Daniela Neculoiu, Diana Țînț

**Affiliations:** ^1^ Faculty of Medicine, “Transilvania” University, Brașov, România; ^2^ Cardiology Department, Cardiovascular Rehabilitation Hospital, “Dr. Benedek Geza”, Covasna, România; ^3^ Cardiology Department, Emergency Clinical County Hospital, Brașov, România; ^4^ Clinical Laboratory Department, Emergency Clinical County Hospital, Brașov, România; ^5^ Cardiology Department, Clinicco Hospital, Brașov, România

**Keywords:** remote ischemic preconditioning, cardioprotection, LOX-1, SOD-1, oxidative stress, coronary artery bypass graft surgery

## Abstract

**Background:**

Coronary artery bypass grafting (CABG) is frequently used to treat severe coronary artery disease (CAD), but it can lead to increased oxidative stress and inflammation, worsening patient outcomes. Remote ischemic preconditioning (RIPC) has been suggested as a potential strategy to protect against these effects by modulating oxidative stress and inflammatory responses, though its impact on specific biomarkers requires further investigation. This study aims to assess the effects of remote ischemic preconditioning on inflammation markers and oxidative stress in patients with severe CAD undergoing coronary artery bypass grafting.

**Methods:**

We conducted a case-control study involving 80 patients with severe coronary artery disease (CAD) scheduled for coronary artery bypass grafting (CABG). Fifty percent of these patients received ischemic preconditioning prior to surgery. Plasma levels of Lectin-like oxidized low-density lipoprotein receptor-1 (LOX-1) and Superoxide dismutase-1 (SOD-1) levels were measured in all individuals using the ELISA method at three important time points: before surgery (visit 1 or V1), immediately post-operatively (visit 2 or V2), and one week post-operatively (visit 3 or V3).

**Results:**

We enrolled 80 patients, of which 40 were assigned to the studied group receiving remote ischemic preconditioning (RIPC) and 40 to the control group. There were no statistically significant differences between the groups regarding baseline, clinical, or operative characteristics. RIPC treatment significantly reduced plasma levels of Lectin-like oxidized low-density lipoprotein (LDL) receptor-1 (LOX-1) (*p* < 0.05) as well as significantly increasing total values of Superoxide dismutase-1 (SOD-1) (*p* < 0.05, respectively). There were notable differences between the studied and control groups at V2 and V3. The studied group had higher SOD-1 levels (*p* < 0.05) and significantly lower LOX-1 levels at both time points (*p* < 0.05).

**Conclusion:**

The significant changes in plasma levels of both LOX-1 and SOD-1 observed in this study strongly suggest that remote ischemic preconditioning (RIPC) plays an important role in reducing oxidative stress and enhancing the antioxidative status of patients. This is evidenced by the marked decrease in LOX-1 levels, alongside a corresponding increase in SOD-1 levels, indicating that RIPC may contribute to improved cardioprotection through these mechanisms.

## 1 Introduction

Remote Ischemic Preconditioning (RIPC) stands as a noninvasive approach designed to protect the heart and other organs from the harmful effects of lethal ischemia and reperfusion injury. The method involves brief cycles of limb ischemia and reperfusion, typically carried out by alternately inflating and deflating a blood pressure cuff on one or more limbs to a suprasystolic pressure value for several cycles (1).

When organs are repeatedly exposed to short-term ischemia-reperfusion, less damage occurs during final reperfusion compared to prolonged ischemic episodes, for example in patients undergoing coronary artery bypass grafting (CABG) and remote ischemic conditioning (RIC) (2, 3).

This method was first described by Murry et al. in 1986 in a study on the heart of dogs (4), which demonstrated the adaptability of the heart to ischemic episodes lasting several minutes, a phenomenon termed ischemic preconditioning (IP) (5, 6).

In humans, Przyklenk et al. described the RIPC phenomena for the first time at the end of the twentieth century (7). After several years, the concept of “cardio protection at a distance” through ischemia conditioning was soon expanded to other tissues and organs, as well as to greater distances from the heart (8). The underlying mechanisms are likely to include transferable humoral release from perfused tissue as well as neuronal responses (9).

Despite myocardial preservation strategies, coronary artery bypass grafting (CABG) is still associated with severe complications.

During CABG, the blood flow to the heart and lungs is temporarily interrupted. Lack of oxygen in these organs disrupts the balance between oxidant and antioxidant enzymes through inefficient degradation of free oxygen radicals, increased/reduced or abnormal activity of antioxidant enzymes, triggering oxidative stress through the accumulation of reactive oxygen species (ROS) (10–12).

Antioxidants are molecules that can inhibit the oxidation of other molecules by destroying oxidants or decreasing the production of free oxygen radicals. The major antioxidant systems present in vascular walls include Superoxide Dismutase (SOD), Catalase, Glutathione Peroxidase, Thioredoxin and Peroxiredoxin (11, 12).

Low-density oxidized lipoprotein receptor type 1 (LOX-1) plays a critical role in the production of atherosclerosis. LOX-1, in addition to binding and internalizing ox-LDL, also contributes to endothelial dysfunction and apoptosis, aiding foam cell formation in macrophages and smooth muscle cells and platelet activation (13).

Remote ischemic preconditioning (RIPC) has been reported to reduce reperfusion injury in people undergoing cardiac surgery and improve clinical outcome (14).

While remote ischemic preconditioning (RIPC) has attracted interest for its potential cardioprotective effects, particularly in patients undergoing cardiac or vascular surgeries, its use is not free from possible complications. Commonly regarded as a non-invasive and low-risk intervention, RIPC may still present challenges in certain patient populations. One possible complication is transient discomfort or pain during the ischemic episodes, which may discourage adherence in some patients (15). Furthermore, there have been reports of hypotension following RIPC, particularly in patients with pre-existing cardiovascular instability (16). This hypotensive response may be more pronounced in patients during general anesthesia or in those with compromised cardiovascular function (17).

Another significant concern is the variability in the efficacy of RIPC, which can be influenced by factors such as age, comorbidities, and medications (especially antihypertensive drugs) (18). For instance, patients with diabetes or hypertension may exhibit an attenuated response to RIPC, possibly due to underlying endothelial dysfunction (19). This reduced responsiveness could limit the protective benefits of the procedure and may increase the risk of ischemic events. Lastly, recent studies have suggested that repetitive RIPC may lead to paradoxical effects, such as exacerbation of ischemia-reperfusion injury in some cases, although this remains an area of ongoing investigation (20).

This study aims to assess the effects of remote ischemic preconditioning on inflammation markers and oxidative stress in patients with severe CAD undergoing coronary artery bypass grafting.

## 2 Materials and methods

### 2.1 Patients

**Figure 1:**
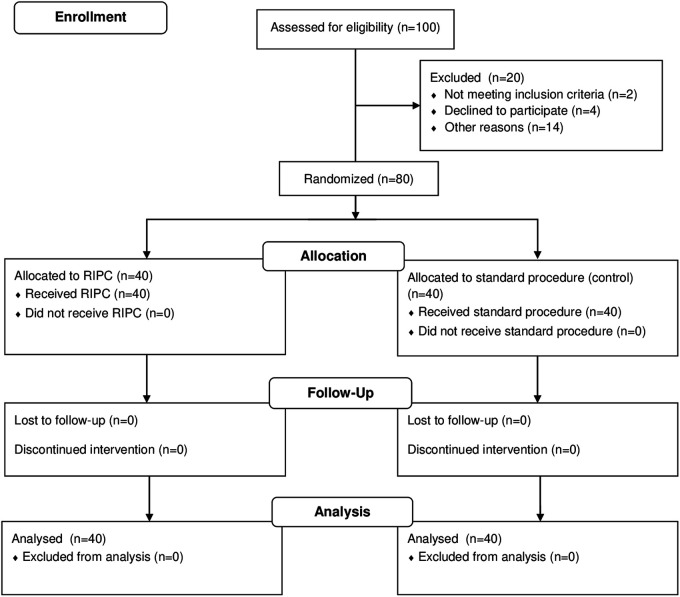
Randomization and follow-up flowchart.

We conducted a prospective observational study, consecutively enrolling 80 adult patients (over 18 years) with severe coronary artery disease who were indicated for elective CABG and consented to undergo the surgery (Figure 1).

The inclusion criteria for this study stipulated that participants must have a body weight greater than 50 kg, be over the age of 18 years, and have provided written informed consent, appropriately dated and signed, prior to their inclusion in the study. Patients with a history of prior revascularization, decompensated heart failure, left ventricular ejection fraction (LVEF) less than 30%, severe renal failure (grade 4 or on dialysis), hepatic dysfunction (Child-Pugh class B or higher), severe pulmonary disease, indication for emergency CABG, and those unable or unwilling to provide informed consent were excluded.

After inclusion, the patients were randomly assigned in a 1:1 ratio to either undergo RIPC or be part of the control group. All members responsible for conducting the study were blinded from the RIPC procedure.

The research was carried out at Clinicco Hospital in Brasov, from January 2020 to November 2022. All procedures in this study were conducted in accordance with the Helsinki Declaration for research involving human subjects and obtained ethical approval from the Ethics Review Board of Transilvania University of Brașov.

Upon admission, patients underwent an initial evaluation comprising their past medical history and present clinical condition, alongside assessments from physical examinations, echocardiography, and laboratory tests.The severity of coronary lesions was quantified using the Syntax score, based on the coronary angiography previously performed in all patients, approximately 2 to 4 weeks prior to the index hospitalization for CABG.

### 2.2 Blood sample collection and analysis

Blood samples for blood cell counts, glycemia, renal function, hepatic function, cardiac biomarkers such as creatin-kinase MB (CK-MB), high sensitivity troponin I (HSTnI), other inflammatory markers and other biological parameters were collected per hospital protocol.

For the specific parameters in our study, we processed the samples by centrifugation to separate the serum from the plasma, which were then labeled and stored at −80 degrees Celsius. We assessed LOX-1 and SOD-1 using the ELISA test in both preconditioned and non-preconditioned populations at three key moments: before surgery (visit 1 or V1), immediately post-operatively (visit 2 or V2), and one week post-operatively (visit 3 or V3).

### 2.3 RIPC procedure

The procedure of RIPC was carried out on the day of surgery, before the induction of anesthesia. It consisted of inflating a blood pressure cuff on the upper and lower limbs to 200 mmHg for 5 min (ischemia stage), followed by a 5-minute time-out with the cuff deflated (reperfusion stage) for a total number of 4 cycles.

Anesthetic management, cardiopulmonary bypass, cardioplegia, surgical techniques, and all other aspects of pre- and postoperative management adhered to the existing protocols in the hospital. All patients received the same types of general anesthesia, including volatile inhalation and intravenous hypnotics.

### 2.4 Statistical analysis

The database has been created using Microsoft Excel 2019 and values have been interpreted by JASP 0.19.0. Categorical variables were given as counts or absolute frequencies. To observe the difference between the mean values of two variables we used Student's *t*-test or Mann-Whitney U (normal distribution or not), and values of *p* < 0.05 were considered statistically significant. *post hoc* analyses, in ANOVA RM, determined the differences within and between groups.

The parameters for the power analysis included an expected effect size of Cohen's d = 0.5 for a medium effect, a significance level (alpha) of 0.05, and a target power of 0.80. This power level was chosen to provide an 80% probability of detecting a true effect, if it exists, thereby balancing the risk of Type I (false positive) and Type II (false negative) errors.

## 3 Results

Of the 100 patients who underwent screening, 80 were successfully enrolled in the study, as depicted in Figure 1. The remaining 20 patients were not included due to the following reasons: 2 patients did not meet the inclusion criteria of a weight above 50 kg, 4 participants declined enrollment in the study due to their unwillingness to sign the informed consent form, and 14 patients were excluded from the study for various reasons, as documented in the CONSORT flow chart: 2 patients presented with severe renal impairment, 2 had a history of prior revascularization, 4 were diagnosed with decompensated heart failure, and 6 faced logistical challenges such as transportation difficulties or residing at a considerable distance from the study site.

From the total of 80 patients that were enrolled, 40 were randomized to receive RIPC, and 40 were allocated to the control group. There were no losses to follow-up, and no patients were prematurely excluded from the study. Additionally, no complications or adverse events were observed during the application of RIPC.

The baseline characteristics of the patients in both the studied and control groups were generally well-matched, with no statistically significant differences observed, as demonstrated in Table 1. The mean age of participants was comparable between the studied group (65.00 ± 7.57 years) and the control group (64.47 ± 8.53 years, *p* = 0.90). Gender distribution also showed a slight difference, with the studied group having a higher proportion of males (85%) compared to the control group (75%), but the difference was not statistically significant. The mean body mass index (BMI) and the smoking status were similar between groups.

**Table 1:** Baseline characteristics of the patients.

	Studied group *N* = 40	Control group *N* = 40	*p*
Age—years (mean) ±SD	65 ± 7.57	64.47 ± 8.53	0.90
Gender (male)—*n*, (%)	34, (85)	29, (72.50)	0.08
BMI—kg/m^2^ ± SD	27.88 ± 4.26	29.36 ± 3.93	0.98
Smoking—*n*, (%)	9, (22.50)	6, (15)	0.08

The clinical characteristics of the patients were generally comparable between the studied and control groups, as illustrated in Table 2. Dyslipidemia was present in all patients in the studied group (100%) compared to 92.5% in the control group (*p* = 0.10). The prevalence of arterial hypertension (HTN) was high in both groups, with 97.5% in the studied group and 92.5% in the control group (*p* = 0.21). Type 2 diabetes mellitus (T2DM) was observed in 40% of the studied group and 37.5% of the control group (*p* = 0.36). A history of myocardial infarction (MI) was noted in 30% of the studied group and 42.5% of the control group (*p* = 0.11), while a history of stroke was identical in both groups at 5% (*p* = 0.98). Peripheral artery disease (PAD) was slightly more prevalent in the control group (15%) compared to the studied group (7.5%), though this difference did not reach statistical significance (*p* = 0.10).

**Table 2:** Clinical characteristics of the patients.

	Studied group *N* = 40	Control group *N* = 40	*p*
Dyslipidemia *n*, (%)	40, (100.00)	37, (92.50)	0.10
HTN *n*, (%)	39, (97.50)	37, (92.50)	0.21
T2DM *n*, (%)	16, (40.00)	15, (37.50)	0.36
Old MI *n*, (%)	12, (30.00)	17, (42.50)	0.11
History of stroke *n*, (%)	2, (5.00)	2, (5.00)	0.98
PAD *n*, (%)	4, (7.50)	8, (15)	0,10

HTN, arterial hypertension; T2DM, type 2 diabetes mellitus; MI, myocardial infarction; PAD, peripheral artery disease.

Overall, the baseline characteristics and clinical profiles of the patients were well-balanced between the two groups, ensuring that the study’s outcomes were not influenced by disparities in these characteristics.

The operative characteristics of the patients exhibited no significant differences between the studied and control groups, suggesting comparable surgical experiences and outcomes across both cohorts, which were not influenced by RIPC, as depicted in as depicted in Table 3. The SYNTAX I score, which assesses the complexity of coronary artery disease, was comparable between the studied group (29.58 ± 7.94) and the control group (30.51 ± 8.59, *p* = 0.61). Aortic clamp time (ACT) and cardiopulmonary bypass time (CPBT) were also similar, with the studied group having an ACT of 65.45 ± 28.20 min compared to 76.42 ± 26.33 min in the control group (*p* = 0.97), and a CPBT of 81.20 ± 28.00 min vs. 93.50 ± 28.31 min (*p* = 0.97). Preoperative left ventricular ejection fraction (LVEF) was slightly higher in the studied group (51.75 ± 7.97%) compared to the control group (48.37 ± 9.63%, *p* = 0.89), with similar postoperative LVEF values (51.62 ± 7.37% vs. 49.37 ± 8.02%, *p* = 0.90). The number of days spent in the intensive care unit (ICU) and the total hospital stay were also closely matched, with ICU stays averaging 3.62 ± 0.86 days in the studied group and 4.10 ± 1.59 days in the control group (*p* = 0.97), and total hospital stays of 10.30 ± 2.51 days and 10.55 ± 3.17 days, respectively (*p* = 0.98).

**Table 3:** Operative characteristics of the patients.

	Studied group *N* = 40	Control group *N* = 40	*p*
SYNTAX I—*n* ± SD	29.58 ± 7.94	30.51 ± 8.59	0.61
ACT (min)—*n* ± SD	65.45 ± 28.20	76.42 ± 26.33	0.97
CPBT (min)—*n* ± SD	81.20 ± 28	93.50 ± 28.31	0.97
LVEF/pre (%)—*n* ± SD	51.75 ± 7.97	48.37 ± 9.63	0.89
LVEF/post (%)—*n* ± SD	51.62 ± 7.37	49.37 ± 8.02	0.90
ICU days (*n*)—*n* ± SD	3.62 ± 0.86	4.10 ± 1.59	0,97
Hosp days (*n*)—*n* ± SD	10.30 ± 2.51	10.55 ± 3.17	0.98

ACT, aortic clamp time; CPBT, cardio-pulmonary bypass time; LVEF/pre, left ventricular ejection fraction preoperatory; LVEF/post, left ventricular ejection fraction postoperatory; ICU, intensive care unit.

**Figure 2:**
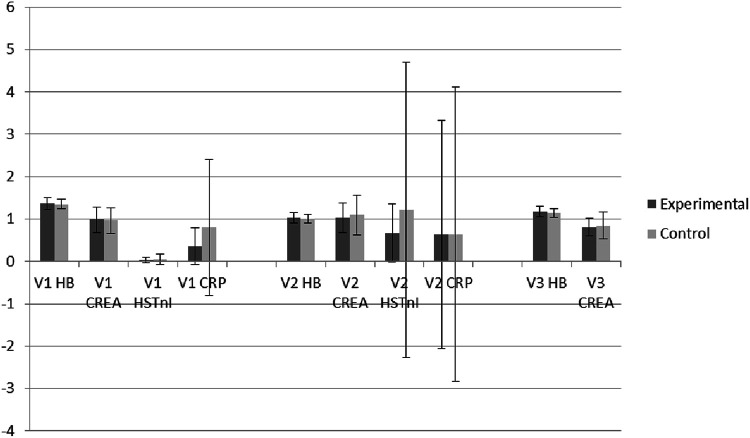
Descriptive box plots regarding general laboratory tests activity.

The general laboratory tests revealed no statistically significant differences between the studied and control groups across all measured parameters, as demonstrated in Table 4 and Figure 2.

**Table 4:** General laboratory tests.

		Studied group *N* = 40	Control group *N* = 40	*p*
V1	Hb—g/dl ± SD	13.66 ± 1.44	13.56 ± 1.19	0.73
	CRP—mg/dl ± SD	0.36 ± 0.43	0.80 ± 1.60	0.10
	HSTnI—pg/ml ± SD	0.03 ± 0.07	0.05 ± 0.12	0.57
	Creatinine—mg/dl ± SD	0.99 ± 0.30	0.97 ± 0.30	0.83
V2	Hb—g/dl ± SD	10.23 ± 1.24	10.14 ± 1.00	0.71
	CRP—mg/dl ± SD	6.35 ± 2.69	6.43 ± 3.47	0.90
	HSTnI—pg/ml ± SD	0.67 ± 0.69	1.22 ± 3.48	0,33
	Creatinine—mg/ml ± SD	1.03 ± 0.35	1.10 ± 0.47	0.43
V3	Hb—g/dl ± SD	11.75 ± 1.28	11.40 ± 1.10	0.19
	Creatinine—mg/ml ± SD	0.81 ± 0.21	0.85 ± 0.32	0.45

Hb, hemoglobine; CRP, C reactive protein; HSTnI, high sensitive troponin I.

Hemoglobin levels (Hb) before surgery were similar between the studied group (13.66 ± 1.44 g/dl) and the control group (13.56 ± 1.19 g/dl, *p* = 0.73), as were levels of C-reactive protein (CRP) (0.36 ± 0.43 mg/dl vs. 0.80 ± 1.60 mg/dl, *p* = 0.10) and high-sensitive troponin I (HSTnI) (0.039 ± 0.076 ng/ml vs. 0.05 ± 0.12 ng/ml, *p* = 0.57).

Serum creatinine levels also showed no significant difference preoperatively between the studied (0.99 ± 0.300 mg/dl) and control groups (0.97 ± 0.30 mg/dl, *p* = 0.83).

Similar trends were observed postoperatively, with Hb (10.23 ± 1.24 g/dl vs. 10.14 ± 1.00 g/dl, *p* = 0.71), CRP (6.35 ± 2.69 mg/dl vs. 6.43 ± 3.47 mg/dl, *p* = 0.90), HSTnI (0.67 ± 0.69 ng/ml vs. 1.22 ± 3.48 ng/ml, *p* = 0.33), and serum creatinine (1.03 ± 0.35 mg/dl vs. 1.10 ± 0.47 mg/dl, *p* = 0.43) with plasma levels remaining comparable between the groups.

Finally, the follow-up measurements at V3 have shown similar laboratory outcomes in both the studied and control groups throughout the study period (Hb 11.75 ± 1.28 g/dl vs. 11.40 ± 1.10 g/dl, *p* = 0.19 and serum creatinine 0.81 ± 0.21 mg/dl vs. 0.85 ± 0.32 mg/dl, *p* = 0.45).

It is important to note that, in accordance with the hospital protocol for laboratory testing, only Hb and creatinine levels were monitored during the follow-up period (Visit 3). Consequently, while the data provided valuable insights into these specific parameters, the absence of follow-up measurements for other laboratory markers, such as CRP and HSTnI, limits the scope of our post-operative analysis.

At the start of the study, a thorough analysis of preoperative biochemical markers was performed, showing that both the studied and control groups had similar plasma levels of SOD-1 and LOX-1 before surgery. This consistency establishes a reliable baseline between the two cohorts, ensuring that any differences in outcomes can be attributed to the interventions rather than to pre-existing variations in these critical markers of oxidative stress and inflammation.

ANOVA analysis of the results of the repeated measures on SOD-1 and LOX-1 levels in patients undergoing coronary artery bypass grafting (CABG) with remote ischemic preconditioning (RIPC) indicated significant differences between the studied and control groups at various time points.

**Figure 3:**
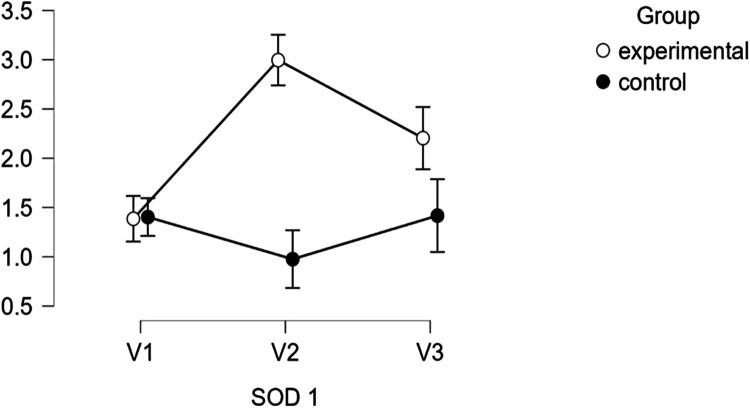
Descriptive plots regarding SOD-1 activity.

For the studied group, SOD-1 levels significantly increased from V1 (1.38 ± 0.20 pg/ml) to V2 (2.99 ± 0.93 pg/ml, *p* < 0.001) and slightly decreased at V3 (2.20 ± 1.22 pg/ml, *p* < 0.01), yet remained elevated compared to baseline. In contrast, the control group exhibited relatively stable SOD-1 levels, with no significant changes between the visits, as illustrated in Table 5 and Figure 3.

**Table 5:** Results of ANOVA RM for SOD-1 and LOX-1 under RIPC on patients undergoing CABG.

	SOD 1		LOX 1	
	Studied (*n* = 40)	Control (*n* = 40)	Studied (*n* = 40)	Control (*n* = 40)
V1	1.38 ± 0.20	1.4 ± 0.24	1,205.50 ± 125.18	1,178.58 ± 257.28
V2	2.99 ± 0.93***	0.97 ± 0.79	427.52 ± 718.44***	604.27 ± 403.41
V3	2.20 ± 1.22**	1.41 ± 1.33	569.99 ± 607.80***	749.36 ± 614.75

*post hoc* Comparisons (Holm correction): between (studied vs. control). SOD-1 and LOX-1 values measured in pg/ml.

*p < 0.05.

**p < 0.01.

***p < 0.001.

**Figure 4:**
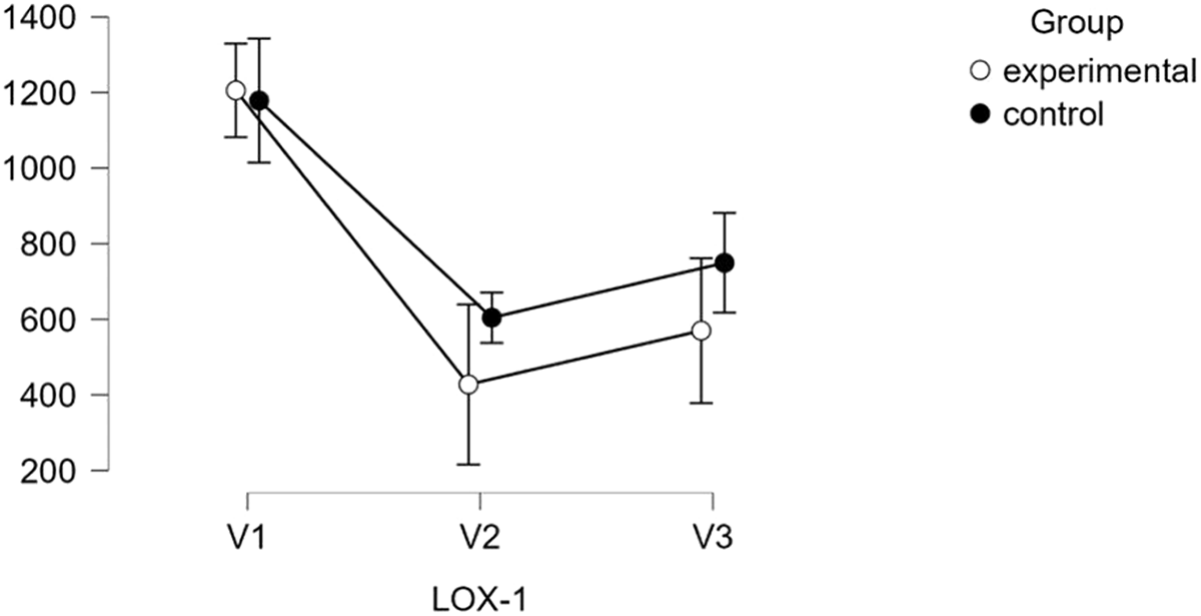
Descriptive plots regarding LOX-1 activity.

LOX-1 levels showed a different trend, with the studied group experiencing a sharp decrease from V1 (1,205.50 ± 125.18 pg/ml) to V2 (427.52 ± 718.44 pg/ml, *p* < 0.001), followed by a partial rebound at V3 (569.99 ± 607.80 pg/ml, *p* < 0.001). The control group, however, showed a gradual increase in LOX-1 levels over time, from V1 (1,178.58 ± 257.28 pg/ml) to V2 (604.27 ± 403.41 pg/ml) and V3 (749.36 ± 614.75 pg/ml), without reaching statistical significance, as demonstrated in Table 5 and Figure 4.

When comparing the studied and control groups, significant differences emerged at V2 and V3 for both SOD-1 and LOX-1 levels. The studied group exhibited higher SOD-1 levels at both V2 and V3 (*p* < 0.01 and *p* < 0.001, respectively), while LOX-1 levels were significantly lower in the studied group at both V2 and V3 (*p* < 0.001).

## 4 Discussion

The findings of our study suggest that RIPC may modulate oxidative stress and inflammatory responses differently between the groups, contributing to the observed differences in biomarker levels over time. These data are consistent with previous research assessing the protective role of RIPC.

LOX-1, a receptor for oxidized low-density lipoprotein (oxLDL), plays a crucial role in the pathogenesis of atherosclerosis and ischemia-reperfusion injury. Elevated levels of LOX-1 are associated with endothelial dysfunction, inflammation, and oxidative stress, which are key factors in the progression of coronary artery disease (CAD) and adverse outcomes during CABG (21).

Our findings align with previous studies that have shown RIPC's ability to reduce oxidative stress markers, although few studies have specifically focused on LOX-1. For example, Li et al. reported that RIPC reduced oxidative damage and improved endothelial function in patients undergoing CABG, though LOX-1 was not directly measured (22). The reduction in LOX-1 observed in our study suggests that RIPC may directly downregulate LOX-1 expression or reduce its activation by decreasing the levels of oxLDL, thereby mitigating oxidative stress and endothelial injury during CABG.

SOD-1 is a critical antioxidant enzyme that converts superoxide radicals into hydrogen peroxide, thus protecting cells from oxidative damage (12). The upregulation of SOD-1 observed in our study is consistent with the known mechanisms of RIPC, which is believed to enhance the endogenous antioxidant defense system.

Previous studies have also demonstrated the ability of RIPC to increase antioxidant enzyme levels, although the specific focus on SOD-1 has been limited. However, comparable findings have been reported in studies involving other antioxidant enzymes. For instance, studies by Heusch et al. and Shimizu et al. observed increased levels of antioxidants and reduced oxidative stress in RIPC-treated patients, suggesting that RIPC exerts a broad effect on the antioxidant defense system (9, 23). The significant increase in SOD-1 levels in our study provides further evidence that RIPC can specifically enhance this critical enzyme's activity, potentially contributing to its cardioprotective effects during CABG.

Regarding the comparative analysis of RIPC effects on LOX-1 and SOD-1, one study by Hagiwara et al. explored the impact of RIPC on oxidative stress markers, including LOX-1 and SOD-1, in a rat model of myocardial ischemia. They found that RIPC significantly decreased LOX-1 expression, which is associated with reduced oxidative stress and endothelial dysfunction. Concurrently, RIPC led to a marked increase in SOD-1 levels, suggesting enhanced antioxidant defenses. These findings indicate that RIPC exerts its cardioprotective effects through a dual mechanism: downregulating pro-oxidative pathways involving LOX-1 and upregulating antioxidant enzymes like SOD-1 (24).

Another study by Ding et al. examined the effects of RIPC on LOX-1 and SOD-1 in a diabetic rat model. This study also reported a decrease in LOX-1 levels and an increase in SOD-1 activity following RIPC, supporting the notion that RIPC can mitigate oxidative stress through modulation of these specific biomarkers. The authors suggested that the decrease in LOX-1 might reduce the pro-inflammatory effects of oxidized LDL, while the increase in SOD-1 could counteract the accumulation of reactive oxygen species (ROS) (25).

The study by Iliodromitis et al. (26) highlights the cardioprotective effects of ischemic preconditioning against myocardial necrosis and apoptosis, both of which are significant concerns during ischemia-reperfusion injury in cardiac surgery. Their research demonstrates that RIPC can mitigate myocardial damage through the activation of various molecular mechanisms, including the reduction of oxidative stress and the inhibition of apoptosis. This is particularly relevant to the current study, which explores the role of antioxidant defense systems, such as SOD-1, and inflamation markers, such as LOX-1, in the context of coronary artery bypass grafting (CABG). The findings from Iliodromitis et al. provide a basis for interpreting how remote ischemic preconditioning (RIPC) might exert similar protective effects in CABG patients by modulating these molecular pathways (26).

While previous research has extensively documented the benefits of RIPC in reducing perioperative myocardial injury and improving clinical outcomes, our findings suggest that the protective mechanisms of RIPC may involve the suppression of pro-oxidative receptors like LOX-1 and the simultaneous upregulation of key antioxidant enzymes like SOD-1.

These findings support the hypothesis that RIPC confers cardioprotection through a multifaceted approach, involving both the attenuation of harmful oxidative processes and the enhancement of protective antioxidant defenses. This dual modulation may be particularly beneficial in the context of CABG, where oxidative stress and inflammation are prevalent and contribute to postoperative complications.

As can be observed in our study also, the methodologies used for determining LOX-1 and SOD-1 in RIPC studies are predominantly centered around ELISA, due to its sensitivity and specificity for detecting these biomarkers in biological fluids. Western blotting and activity assays also play important roles, particularly for tissue-specific studies and functional assessments (27). The selection of methodology depends on the study's focus, whether it is quantifying protein levels or assessing enzymatic activity.

### 4.1 Implications and future directions

Our results contribute to the growing body of evidence that RIPC can modulate oxidative stress and inflammatory pathways, thereby offering potential cardioprotective benefits during surgical interventions like CABG. The results of our study have important clinical implications, suggesting that RIPC could be a valuable strategy to improve outcomes in patients undergoing CABG by targeting specific molecular pathways involved in oxidative stress and inflammation. Future studies should aim to confirm these findings in larger, multicenter trials and explore the mechanistic basis of RIPC's effects on LOX-1 and SOD-1 in greater detail. Additionally, the potential of RIPC as an adjunct therapy to pharmacological interventions targeting oxidative stress in CABG patients warrants further investigation.

### 4.2 Study limitations

This study has several limitations that should be considered. First, the sample size was relatively small, which may limit the generalizability of the findings, as demonstrated in Table 6.

**Table 6:** *Post-hoc* power analysis.

Power	Without RIPC	With RIPC	Cohen's |δ|	α
0.598	40	40	0.500	0.050
Power by effect size				
True effect size		Power to detect		Description
0 < |δ| ≤ 0.444		≤50%		Likely miss
0.444 < |δ| ≤ 0.634		50%–80%		Good chance of missing
0.634 < |δ| ≤ 0.816		80%–95%		Probably detect
|δ| ≥ 0.816		≥95%		Almost surely detect

RIPC, remote ischemic preconditioning.

The post-hoc power analysis presented in Table 6 highlights the limitation of this study stated above. With a sample size of 40 participants per group, the statistical power achieved was 0.598, which falls below the conventional threshold of 0.80, indicating a moderate risk of Type II error. The effect size analysis further underscores this concern, as the study was adequately powered (≥95%) only to detect large effect sizes (|δ| ≥ 0.816). For medium effect sizes (0.634 < |δ| ≤ 0.816), the power ranged between 80% and 95%, suggesting a reasonable likelihood of detecting differences, though still with some risk of underestimation. However, for smaller effect sizes (|δ| ≤ 0.634), the power drops considerably, indicating a substantial chance of missing significant effects. This limitation suggests that the study may not be sufficiently sensitive to detect smaller, yet potentially clinically relevant, differences between groups.

Additionally, the study was conducted in a single center, potentially introducing selection bias. The short-term follow-up period did not allow for the assessment of long-term effects of RIPC on LOX-1 and SOD-1 levels post-CABG. Finally, while we observed significant changes in these biomarkers, the underlying mechanisms of how RIPC modulates LOX-1 and SOD-1 remain unclear, warranting further studies.

## 5 Conclusion

The significant changes in plasma levels of both LOX-1 and SOD-1 observed in this study strongly suggest that remote ischemic preconditioning (RIPC) plays an important role in reducing oxidative stress and enhancing the antioxidative status of patients. This is evidenced by the marked decrease in LOX-1 levels, alongside a corresponding increase in SOD-1 levels, indicating that RIPC may contribute to improved cardioprotection through these mechanisms.

## Data Availability

The raw data supporting the conclusions of this article will be made available by the authors, without undue reservation.

## References

[1] Lang JA Kim J. Remote ischaemic preconditioning - translating cardiovascular benefits to humans. J Physiol. (2022) 600(13):3053–67. 10.1113/JP282568 35596644 PMC9327506

[2] Laurikka J Wu Z-K Iisalo P. Regional ischemic preconditioning enhances myocardial performance in off-pump coronary artery bypass grafting. Chest. (2002) 121(4):1183–9. 10.1378/chest.121.4.1183 11948051

[3] Gunata M Parlakpinar H. A review of myocardial ischaemia/reperfusion injury: pathophysiology, experimental models, biomarkers, genetics and pharmacological treatment. Cell Biochem Funct. (2021) 39(2):190–217. 10.1002/cbf.3587 32892450

[4] Murry CE Jennings RB Reimer KA. Preconditioning with ischemia: a delay of lethal cell injury in ischemic myocardium. Circulation. (1986) 74(5):1124–36. 10.1161/01.CIR.74.5.1124 3769170

[5] Liu GS Stanley AW Downey JM. Ischaemic preconditioning is not dependent on neutrophils or glycolytic substrate at reperfusion in rabbit heart. Cardiovasc Res. (1992) 26(12):1195–8. 10.1093/cvr/26.12.1195 1288866

[6] Vahlhaus C Schulz R Post H Rose J Heusch G. Prevention of ischemic preconditioning only by combined inhibition of protein kinase C and protein tyrosine kinase in pigs. J Mol Cell Cardiol. (1998) 30(2):197–209. 10.1006/jmcc.1997.0609 9514996

[7] Przyklenk K Bauer B Ovize M Kloner RA Whittaker P. Regional ischemic “preconditioning” protects remote virgin myocardium from subsequent sustained coronary occlusion. Circulation. (1993) 87:893–9. 10.1161/01.CIR.87.3.893 7680290

[8] Heusch G. 25 Years of remote ischemic conditioning: from laboratory curiosity to clinical outcome. Basic Res Cardiol. (2018) 113:15. 10.1007/s00395-018-0673-2 29516255

[9] Heusch G Botker HE Przyklenk K Redington A Yellon D. Remote ischemic conditioning. J Am Coll Cardiol. (2015) 65:177–95. 10.1016/j.jacc.2014.10.031 25593060 PMC4297315

[10] Bielli A Scioli MG Mazzaglia D Doldo E Orlandi A. Antioxidants and vascular health. Life Sci. (2015) 143:209–16. 10.1016/j.lfs.2015.11.012 26585821

[11] Poznyak AV Grechko AV Orekhova VA Chegodaev YS Wu WK Orekhov AN. Oxidative stress and antioxidants in atherosclerosis development and treatment. Biology (Basel). (2020) 9:60. 10.3390/biology9030060 32245238 PMC7150948

[12] Wassmann S Wassmann K Nickenig G. Modulation of oxidant and antioxidant enzyme expression and function in vascular cells. Hypertension. (2004) 44:381–6. 10.1161/01.HYP.0000142232.29764.a7 15337734

[13] Chen M Nagase M Fujita T Narumiya S Masaki T Sawamura T. Diabetes enhances lectin-like oxidized LDL receptor-1 (LOX-1) expression in the vascular endothelium: possible role of LOX-1 ligand and AGE. Biochem Biophys Res Commun. (2001) 287:962–8. 10.1006/bbrc.2001.5674 11573959

[14] Benstoem C Stoppe C Liakopoulos OJ Ney J Hasenclever D Meybohm P Remote ischaemic preconditioning for coronary artery bypass grafting (with or without valve surgery). Cochrane Database Syst Rev. (2017) 5:CD011719. 10.1002/14651858.CD011719.pub3 28475274 PMC6481544

[15] Heusch G. Molecular basis of cardioprotection: signal transduction in ischemic pre-, post-, and remote conditioning. Circ Res. (2015) 116(4):674–99. 10.1161/CIRCRESAHA.116.305348 25677517

[16] Candilio L Malik A Hausenloy DJ Yellon DM. Protection from ischaemia–reperfusion injury: a review of the experimental and clinical evidence for ischaemic preconditioning and postconditioning. Br Med Bull. (2013) 106(1):211–21. 10.1093/bmb/ldt040

[17] Sloth AD Schmidt MR Munk K Kharbanda RK Redington AN Schmidt M Improved long-term clinical outcomes in patients with ST-elevation myocardial infarction undergoing remote ischemic conditioning as an adjunct to primary percutaneous coronary intervention. Eur Heart J. (2014) 35(3):168–75. 10.1093/eurheartj/eht369 24031025

[18] Hansen J Myrmel T Andressen K Mjølstad OC Kirkebøen KA. Remote ischemic preconditioning: from experimental evidence to clinical translation. Br J Anaesth. (2021) 126(1):32–42. 10.1016/j.bja.2020.10.017

[19] Loukogeorgakis SP Panagiotidou AT Broadhead MW Donald A Deanfield JE Macallister RJ. Remote ischemic preconditioning provides early and late protection against endothelial ischemia-reperfusion injury in humans: role of the autonomic nervous system. J Am Coll Cardiol. (2005) 46(3):450–6. 10.1016/j.jacc.2005.04.044 16053957

[20] Zhou C Pan R Liu X. Paradoxical effects of ischemic conditioning: more questions than answers. Int J Mol Sci. (2020) 21(1):121. 10.3390/ijms21010121

[21] Sugano Y Anzai T Yoshikawa T Satoh T Nomura T Suzuki Ogawa S. Oxidized low-density lipoprotein receptor LOX-1 plays a crucial role in the pathogenesis of atherosclerosis and vasospasm-induced myocardial infarction in dogs. Circ Res. (2002) 90(2):182–8. 10.1161/hh0202.103940 11834711

[22] Li C Li YS Xu M Wen SH Yao X Wu Y Effects of remote ischemic preconditioning on endothelial function and oxidative stress in patients undergoing cardiopulmonary bypass. Ann Thorac Surg. (2013) 96(3):844–51. 10.1016/j.athoracsur.2013.04.075 23810177

[23] Shimizu M Tropak M Diaz RJ Suto F Surendra H Kuzmin E Transient limb ischemia remotely preconditions through a humoral mechanism acting directly on cardiomyocytes: evidence suggesting cross-species protection. Clin Sci (Lond). (2009) 117(5):191–200. 10.1042/CS20080523 19175358

[24] He K Chen X Han C Xu L Zhang J Zhang M Lipopolysaccharide-induced cross-tolerance against renal ischemia-reperfusion injury is mediated by hypoxia-inducible factor-2α-regulated nitric oxide production. Kidney Int. (2014) 85(2):276–88. 10.1038/ki.2013.342 24025643

[25] Li Q Ding J Xia B Liu K Zheng K Wu J L-theanine alleviates myocardial ischemia/reperfusion injury by suppressing oxidative stress and apoptosis through activation of the JAK2/STAT3 pathway in mice. Mol Med. (2024) 30(1):98. 10.1186/s10020-024-00865-0 38943069 PMC11214244

[26] Iliodromitis EK Lazou A Kremastinos DT. Ischemic preconditioning: protection against myocardial necrosis and apoptosis. Vasc Health Risk Manag. (2007) 3(5):629–37. 18078014 PMC2291307

[27] Kattoor AJ Goel A Mehta JL. LOX-1: regulation, signaling and Its role in atherosclerosis. Antioxidants (Basel). (2019) 8(7):218. 10.3390/antiox8070218 31336709 PMC6680802

